# Cytokinesis in *fra2 Arabidopsis thaliana* p60-katanin Mutant: Defects in Cell Plate/Daughter Wall Formation

**DOI:** 10.3390/ijms22031405

**Published:** 2021-01-30

**Authors:** Emmanuel Panteris, Anna Kouskouveli, Dimitris Pappas, Ioannis-Dimosthenis S. Adamakis

**Affiliations:** 1 Department of Botany, School of Biology, Aristotle University of Thessaloniki, 541 24 Thessaloniki, Greece; akouskou@bio.auth.gr (A.K.); dtpappas@bio.auth.gr (D.P.); 2 Section of Botany, Department of Biology, National and Kapodistrian University of Athens, 157 84 Athens, Greece

**Keywords:** *Arabidopsis thaliana*, callose, cell plate, cross wall, cytokinesis, homogalacturonans, katanin, KNOLLE syntaxin, microtubules, phragmoplast

## Abstract

Cytokinesis is accomplished in higher plants by the phragmoplast, creating and conducting the cell plate to separate daughter nuclei by a new cell wall. The microtubule-severing enzyme p60-katanin plays an important role in the centrifugal expansion and timely disappearance of phragmoplast microtubules. Consequently, aberrant structure and delayed expansion rate of the phragmoplast have been reported to occur in p60-katanin mutants. Here, the consequences of p60-katanin malfunction in cell plate/daughter wall formation were investigated by transmission electron microscopy (TEM), in root cells of the *fra2* *Arabidopsis thaliana* loss-of-function mutant. In addition, deviations in the chemical composition of cell plate/new cell wall were identified by immunolabeling and confocal microscopy. It was found that, apart from defective phragmoplast microtubule organization, cell plates/new cell walls also appeared faulty in structure, being unevenly thick and perforated by large gaps. In addition, demethylesterified homogalacturonans were prematurely present in *fra2* cell plates, while callose content was significantly lower than in the wild type. Furthermore, KNOLLE syntaxin disappeared from newly formed cell walls in *fra2* earlier than in the wild type. Taken together, these observations indicate that delayed cytokinesis, due to faulty phragmoplast organization and expansion, results in a loss of synchronization between cell plate growth and its chemical maturation.

## 1. Introduction

Cytokinesis, the process by which parent cells divide after karyokinesis, is accomplished in higher plants by the function of the phragmoplast [1,2,3]. Typically, an array of two anti-parallel microtubule groups, originally deriving by the central spindle, mediates the formation of a cell plate, consisting of fusing dictyosome vesicles [3,4]. These microtubules are perpendicular to the cell plate, with their plus ends pointing toward the equatorial region [5]. Both the phragmoplast and cell plate initiate between the separated chromosome groups at telophase, then expand centrifugally to meet the parent cell wall at the cortical division site [3,4,5]. Cell plate is built by dictyosome-derived vesicles, the fusion of which into a unified cell plate is mediated by KNOLLE, a cytokinesis-specific plant syntaxin that, apart from being recruited to the cell plate, also persists for a while on newly built cell walls [6,7]. Initially, cell plate consists mainly of callose, offering integrity and rigidity, and highly methylesterified pectins, while a milestone of its maturation is the replacement of callose by cellulose and the de-esterification of pectins [8]. During cell plate expansion, phragmoplast microtubules depolymerize in the center, where the cell plate is stabilized, and new ones appear at the rim of the expanding cell plate, where new vesicles are added [4]. After expansion outside the daughter nuclei zone and initial cell plate stabilization, phragmoplast microtubules are typically short, perpendicular to the cell plate, eventually disappearing after the final fusion of the cell plate with parent cell walls [9].

Several proteins are involved in phragmoplast organization, expansion, and function, among which p60-katanin, a microtubule-severing enzyme that belongs in the family of AAA ATPases [10]. In particular, p60-katanin severs microtubules at their (-) ends, releasing them from their nucleation sites and allowing them to achieve their appropriate organization pattern [11]. Initially, failure in severing by p60-katanin was associated with loss of cell growth anisotropy, due to cortical microtubule disorientation, a defect observed in a variety of mutants [12,13,14,15]. Furthermore, microtubule severing by p60-katanin was found to be important in several developmental processes of plants [16], both vegetative and reproductive [17], affecting tissue patterning [18]. During cytokinesis, p60-katanin was found to be located at the (-) microtubule ends of the phragmoplast, distal to the cell plate [19], most probably mediating their release from the nuclear surface and “trimming” the expanding phragmoplast microtubules to the appropriate length and arrangement [19]. As already reported by confocal microscopy observations, failure in microtubule severing by p60-katanin, in the relevant *Arabidopsis thaliana* mutants, results in a “double-arrow” phragmoplast configuration: during expansion, microtubules appear elongated and bent, as their (-) ends may remain attached to the nuclear surface [20]. This structural aberration also affects the rate of cytokinesis, which in p60-katanin mutants is significantly slower than in the wild-type [21].

Although the above defects in phragmoplast organization and function are well-established, the fine structure of cytokinesis in p60-katanin mutants has not been studied yet. Therefore, in an effort to further elucidate the role of p60-katanin in cytokinesis, a detailed structural investigation of cell plate/daughter wall formation was performed in *fra2 A. thaliana* p60-katanin mutant by transmission electron microscopy (TEM). Moreover, the pectic components of the developing cell plate and cross wall were identified by a monoclonal antibody (JIM5), while detection of callose and the KNOLLE syntaxin was also performed by epi-fluorescence and/or confocal microscopy.

## 2. Results

### 2.1. Cell Plate Formation in the Wild-Type

**Figure 1:**
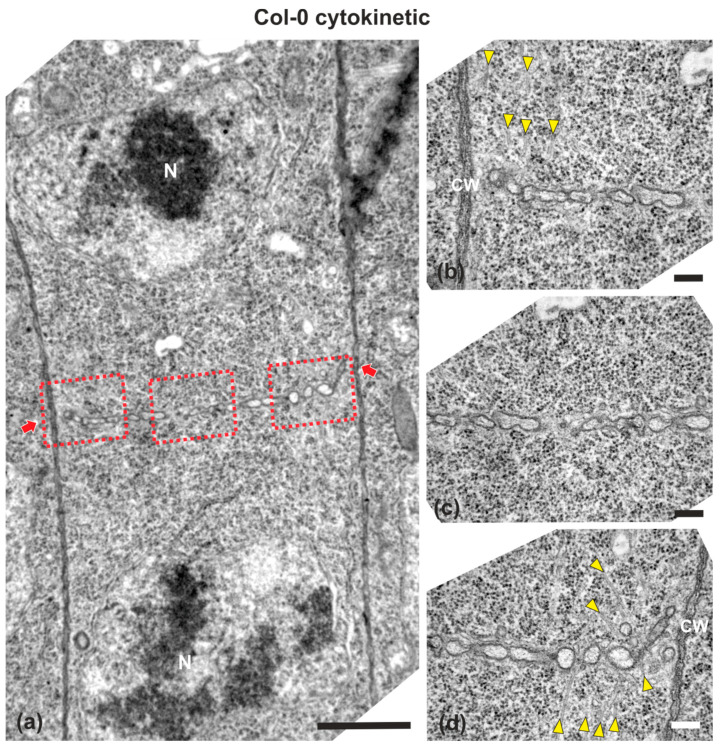
TEM micrographs of a cytokinetic wild-type root cell at central longitudinal section. (**a**) Lower magnification view of the whole cell. The daughter nuclei (N) exhibit telophase morphology, baring condensed chromatin. (**b**–**d**) Higher magnification images of the corresponding areas, defined by rectangles in (**a**). The cell plate (defined by arrows in a) is built up by vesicles, not yet connected with the parent wall at its edges, where phragmoplast microtubules can be observed ((**b**,**d**), arrowheads). Microtubules are absent from the central cell plate part (**c**). CW, cell wall. Bars: (**a**), 5 μm; (**b**–**d**), 200 nm.

**Figure 2:**
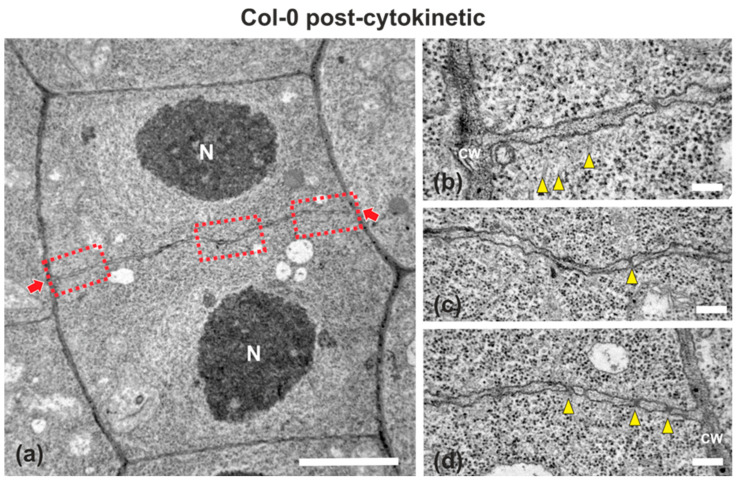
TEM micrographs of a post-cytokinetic wild-type root cell at central longitudinal section. (**a**) Lower magnification view of the whole cell. The daughter nuclei (N) exhibit interphase morphology. (**b**–**d**) Higher magnification images of the corresponding areas, defined by rectangles in (**a**). The mature cell plate/nascent cross wall (defined by arrows in (**a**)) is consolidated, connected with the parent wall, exhibiting uniform width, with some plasmodesmata (arrowheads in (**c**,**d**)) interrupting its continuity. Some remnants of phragmoplast microtubules can be still observed ((**b**), arrowheads). CW, cell wall. Bars: (**a**), 5 μm; (**b**–**d**), 200 nm.

**Figure 3:**
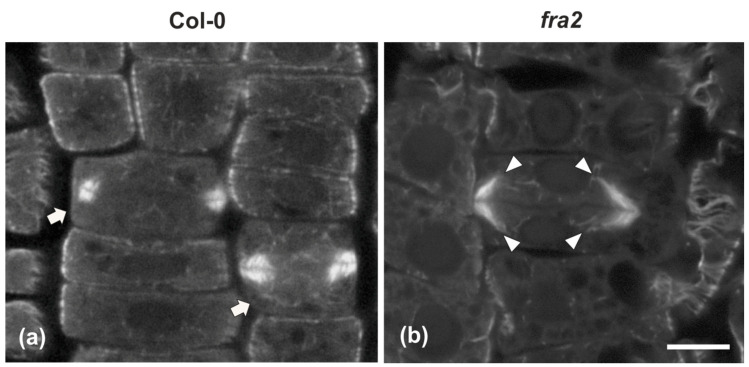
Single confocal laser scanning microscope (CLSM) sections depicting wild-type ((**a**), arrows) and *fra2* (**b**) cytokinetic cells after tubulin immunostaining. While the expanding phragmoplast of wild-type cell (**a**) consists of short microtubules, restricted at the edges of the cell plate, those of *fra2* ((**b**), arrowheads) cells are long, bended, and extended towards the daughter nuclei, exhibiting a “double-arrow” shape. Scale bar 10 μm.

Wild-type (ecotype Columbia; Col-0) root cells at cytokinesis displayed typical cell plates at each stage of development (early, mid and late) [3], with the daughter nuclei being well-separated (Figure 1 and Figure 2). In the above cells, after the formation of a cell plate assembly matrix at the center of the cell, cell plates, uniform in thickness (Figure 1 and Figure 2), expanded centrifugally to the parental cell wall, where they fused, thus successfully completing cell division (Figure 2). The nascent cross wall was also uniformly thick, interrupted only by primary plasmodesmata (Figure 2). Simultaneously with cell plate maturation in the central part of the cell, phragmoplast microtubules disassembled in this area, expanding centrifugally and remaining restricted to the growing edge of the cell plate (Figure 3a). Once the cell plate reached and fused with the parent cell wall, phragmoplast microtubules disappeared (Figure 2).

### 2.2. Cell Plate Formation in fra2

**Figure A1:**
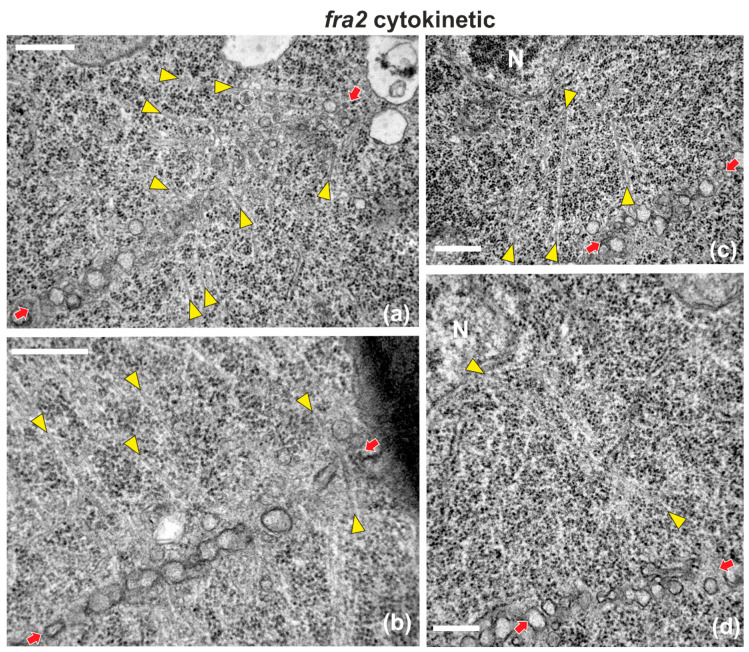
Selected TEM micrographs of cytokinetic *fra2* root cells, exhibiting a variety of deviations in microtubule organization. Phragmoplast microtubules are pointed by yellow arrowheads, while cell plates are defined by red arrows in all micrographs. (**a**) Phragmoplast microtubules at the expanding edge of the cell plate are bended, forming an acute angle with it. Some of them appear branched (branch point inside the white circle). (**b**) A microtubule penetrating through the whole cell plate width can be observed at the right part (beside the right arrow) (**c**,**d**) Phragmoplast microtubules extend from the cell plate rim to the nuclear (N) surface (see also Figure 4). Bars: 500 nm.

While in cytokinetic root cells of the wild-type, with a growing cell plate, the microtubules of the expanding phragmoplast were short and restricted at the edges of the cell plate (Figure 3a), in cytokinetic root cells of *fra2*, at a stage similar to that of the wild-type, phragmoplast microtubules were mainly restricted at the edges of the growing cell plate, but they were longer than those of the phragmoplasts of wild-type cells, bending and extending towards the daughter nuclei, having a “double arrow” configuration at side view (Figure 3b, Figure A1a), as already reported [19,20].

**Figure 4:**
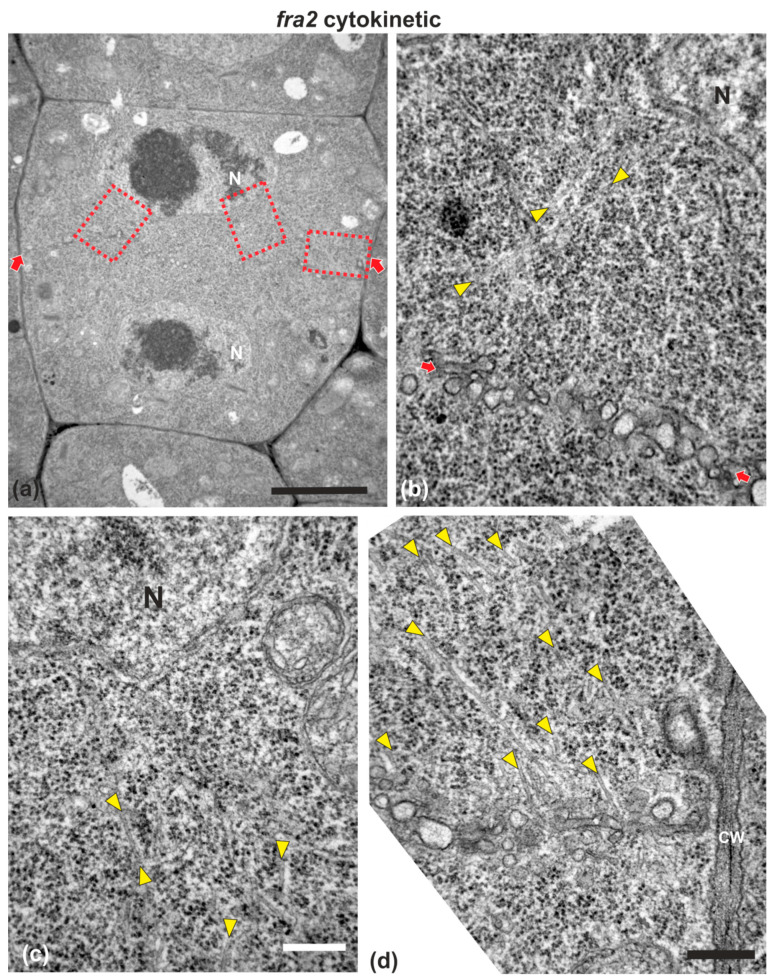
TEM micrographs of a cytokinetic *fra2* root cell at central longitudinal section. (**a**) Lower magnification view of the whole cell. The daughter nuclei (N) exhibit telophase morphology with condensed chromatin. (**b**–**d**) Higher magnification images of the corresponding areas, defined by rectangles in (**a**). Numerous phragmoplast microtubules (arrowheads) appear bending towards the nucleus, some of which extending up to the nuclear surface (**b**,**c**). The cell plate (defined by arrows in (**a**)) consists of numerous unaligned vesicles (arrows in (**b**); compare with the aligned cell plate vesicles in the wild-type in Figure 1). CW, cell wall. Bars: (**a**), 5 μm; (**b**–**d**), 200 nm.

**Figure 5:**
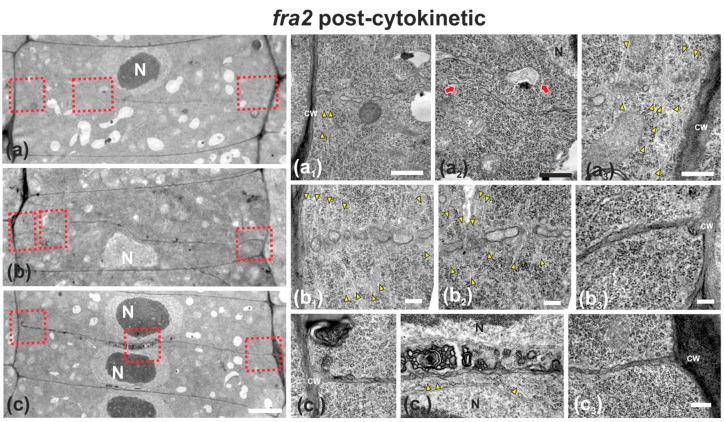
TEM micrographs of post-cytokinetic *fra2* root cells. (**a**–**c**): Lower magnification view of the whole cells. The daughter nuclei depicted (N) exhibit interphase morphology (**a**–**c**). Higher magnification images of the corresponding areas, defined by rectangles in a–c. (**a_1_**–**a_3_**): The cell plate/cross wall is unevenly consolidated, still not connected with the parent wall at the right part (**a_3_**), where phragmoplast microtubules still persist (**a_3_**, arrowheads). At the left part (**a_1_**) the cross wall, though connected to the parent wall, is highly perforated. Close to the nucleus (**a_2_**) the cross wall appears unevenly thick and discontinuous (**a_2_**, arrows), also baring large gaps. (**b_1_**–**b_3_**): Cell plate/cross wall is not consolidated at the left side (**b_1_**,**b_2_**), though connected with the parent wall at the right (**b_3_**). Numerous phragmoplast microtubules (**b_1_**, **b_2_**, arrowheads) are still prominent. (**c_1_**–**c_3_**): The cross wall is consolidated, connected with the parent wall at the left (**c_1_**) and right (**c_3_**). At its central part, large multilamellar bodies can be observed in touch with the cell plate, while arrows point to microtubules (**c_2_**). CW, cell wall. Bars: (**a**–**c**), 5 μm; (**a_1_**–**c_3_**), 200 nm.

**Figure 6:**
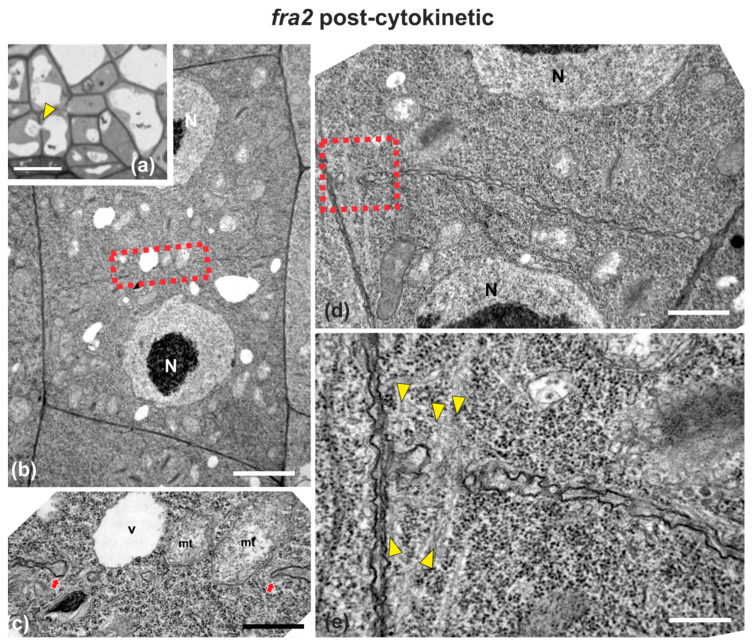
Light (**a**) and TEM (**b**–**e**) micrographs of post-cytokinetic *fra2* root cells. (**a**) Light micrograph of root cap cells, exhibiting a cell wall (arrowhead) with a large gap, with a vacuole penetrating through it. (**b**,**d**) Lower magnification view of whole cells. The daughter nuclei (N) exhibit interphase morphology. (**c**) Higher magnification of the area defined by rectangle in (**b**): Cross wall gap (arrows) with a vacuole (V) and mitochondria (mt) almost penetrating through it. (**e**) Higher magnification of the area defined by rectangle in (**d**): Cross wall gap with microtubules (arrowheads) penetrating through it. CW, cell wall. Bars: (**a**), 5 μm; (**b**,**d**), 2 μm, (**c**,**e**), 200 nm.

In *fra2* roots, cell plates of cytokinetic cells exhibited several structural defects. Early cell plates of mid-cytokinetic cells exhibited asymmetrical, unilateral growth (Figure 4), while in many cases the phragmoplast microtubules extended towards the daughter nuclei (Figure 4b–d, Figure A1c,d), an observation in accordance with observations with tubulin immunostaining (see Figure 3b). In addition, in several cases, phragmoplast microtubules were observed to penetrate through the whole cell plate width, “protruding” into the neighboring cytoplasm (Figure A1b). In post-cytokinetic cells, cross walls were discontinuous, consisting of still growing cell plate parts (Figure 5a–b_3_) and unilaterally matured cross wall fragments (Figure 5a_2_). In some cases, the cross walls were abnormally developed, lined by aggregations of multilamellar bodies (Figure 5c–c_3_), most probably representing remnants of vesicle membranes. Phragmoplasts persisted in post-cytokinetic cells, exhibiting numerous microtubules (Figure 5b–b_3_). Moreover, post-cytokinetic cell walls exhibited extensive gaps (Figure 6) or persisting phragmoplast microtubules penetrating through the newly-formed cell wall (Figure 6).

### 2.3. Callose, KNOLLE, and Demethylesterified Homogalacturonan (DeSPHG) Localization in Cell Plates of Wild-Type and fra2

**Figure 7:**
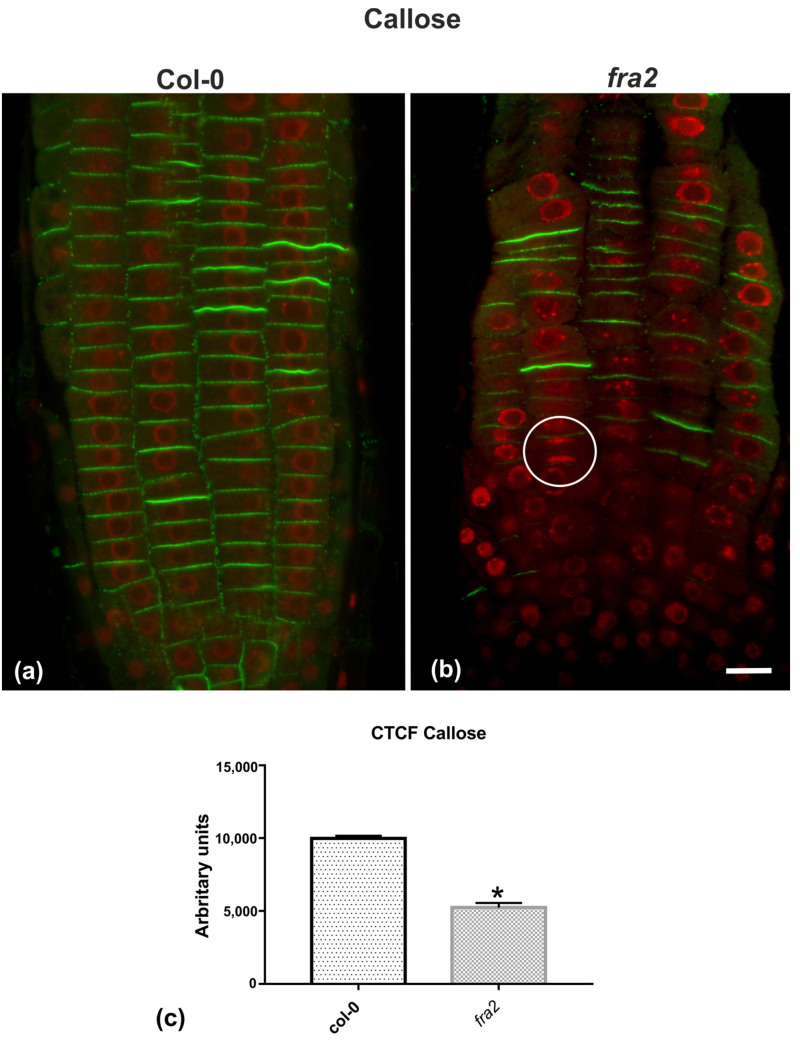
(**a**,**b**) Single CLSM sections depicting callose (anti-*β*-1,3-glucan) localization (green) in root cells of wild-type (**a**) and *fra2* (b). DNA counterstaining appears in red (pseudocolor). (**c**) Graph illustrating the corrected total cell fluorescence (CTCF) intensity measurements of callose (anti-*β*-1,3-glucan) localization in wild-type and *fra2*. The newly created cell walls of wild-type root cells are intensively stained with the anti-*β*-1,3-glucan antibody (**a**), while in *fra2* root cytokinetic cells it exhibits a reduced signal (**b**), or appears occasionally absent (not any callose signal between sister chromosome groups in the white circle). (**c**) CTCF is statistically significantly reduced (asterisk, * *p* < 0.05) in *fra2* root cells. Bar: 10 μm.

**Figure 8:**
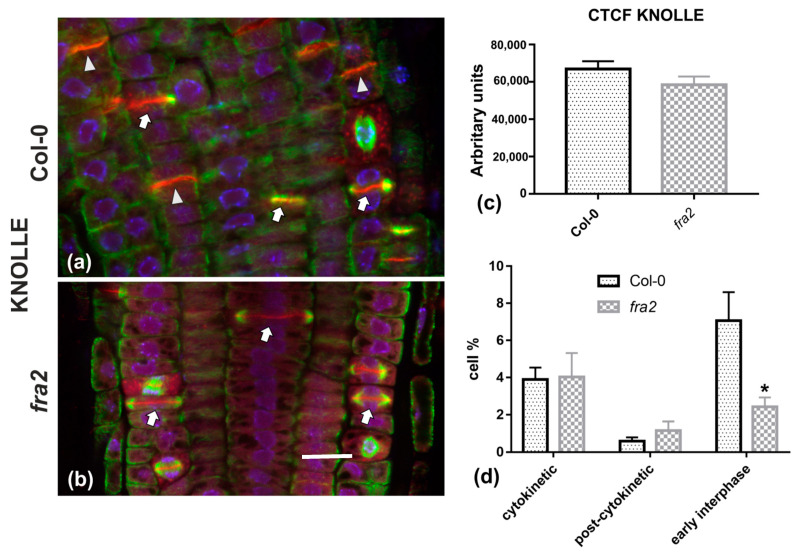
(**a**,**b**) Single CLSM sections depicting anti-KNOLLE (red) and anti-*α*-tubulin (green) localization in root cells of wild-type (**a**) and *fra2* (**b**). DNA counterstaining appears in blue. (**c**) Graph illustrating the corrected total cell fluorescence (CTCF) intensity measurements of anti-KNOLLE localization in wild-type and *fra2*. (**d**) Graph illustrating the cytokinetic, post-cytokinetic, and early interphase cell percentage counts exhibiting anti-KNOLLE signal in wild type and *fra2.* (**a**,**b**): The developing cell plates (arrows in a, b) in both wild-type and *fra2* and the newly developed cell walls (arrowheads in a) of wild-type root cells are intensely stained with the anti-KNOLLE antibody. (**c**,**d**) CTCF of KNOLLE is not statistically significantly reduced (* *p* > 0.05) in *fra2* root cells, however, it is statistically significantly reduced (asterisk in (**d**); * *p* < 0.05) in *fra2* early interphase cells. Bar: 10 μm.

Given the importance of callose for cell plate establishment and integrity [8,22], its distribution was investigated by immunolocalization. Confocal laser scanning microscope (CLSM) observations revealed a stronger and more widespread signal in meristematic root cells of the wild-type (Figure 7a), in comparison with the mutant (Figure 7b), which was also confirmed by fluorescence intensity measurements (Figure 7c). In particular, callose signal was unexpectedly low or occasionally absent, even in cytokinetic cells with developing cell plates (Figure 7b). The distribution of KNOLLE was also followed by CLSM after immunolocalization, since this syntaxin appears pivotal for higher plant cytokinesis [6]. KNOLLE was found in dividing cells of both wild-type and mutant roots, with a prominent signal on cell plates of all cytokinetic cells, nascent cross walls of all post cytokinetic cells, and the transverse cell wall of certain interphase cells (Figure 8). However, interphase cells with a visible KNOLLE signal at the transverse cell walls were significantly fewer in the mutant, compared to the wild type (Figure 8d).

**Figure 9:**
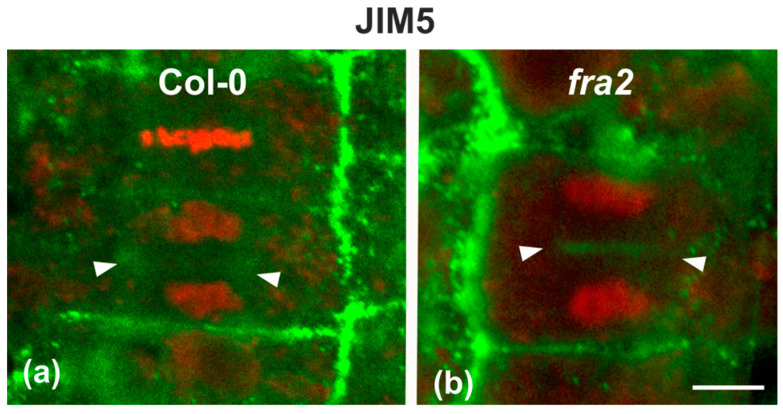
Single CLSM sections depicting DeSPHG localization by the JIM5 (green) antibody in cytokinetic root cells of wild-type (**a**) and *fra2* (**b**). DNA counterstaining appears in red (pseudocolor). The developing cell plate (defined by arrowheads) of wild-type root cells is poorly stained with the JIM5 antibody (**a**), while in *fra2* it exhibits a stronger JIM5 signal ((**b**), arrowheads). Bar: 10 μm.

**Figure 10:**
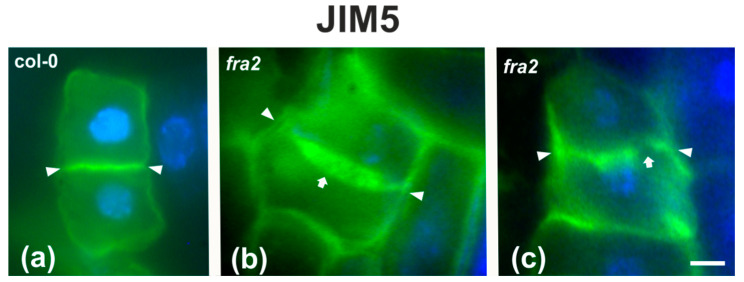
DeSPHG localization by the JIM5 (green) antibody in post-cytokinetic cells of wild-type (**a**) and *fra2* (**b**,**c**) roots. DNA counterstaining appears blue. JIM5 fluorescence reveals that the consolidated cross wall (defined by arrowheads in all figures) is evenly thick in the wild-type (**a**), while in *fra2* it appears unevenly thick (arrow in (**b**)) or with gaps (arrow in (**c**)). Bars: 10 μm.

Since the presence of demethylesterified pectins may be used as an indicator for the degree of cell plate/cross wall maturation [8], demethylesterified homogalaturonans (DeSPHGs) were traced in cytokinetic and post-cytokinetic root cells by JIM5 (anti-DeSPHG antibody) immunolocalization. In cells with newly formed cell plates, JIM5 signal was present in both *fra2* and the wild type. However, cell plates of cytokinetic *fra2* root cells were more strongly labeled (Figure 9b), compared to those of wild-type cells at a similar stage, where JIM5 localization appeared weaker (Figure 9a; cf. Figure 9b). In post-cytokinetic cells, nascent cross walls were also labeled by JIM5 signal (Figure 10). However, in *fra2* JIM5 distribution was uneven (Figure 10b,c; cf. Figure 10a).

## 3. Discussion

According to our observations, p60-katanin malfunction results in defective cell plate formation and maturation. The observed defects can be distinguished as structural (shape, width, integrity) and chemical (polysaccharide distribution). Apart from confirming the defective phragmoplast morphology [19,20] and expansion rate [21], already reported by CLSM studies, the data presented here offer a deeper view in the cytokinetic defects commonly found in p60-katanin mutants.

Delayed phragmoplast/cell plate expansion [21] may well be the cause for the difference in cell plate contents. It is well-established that cell plate vesicles initially contain highly esterified homogalacturonans, while demethylesterified homogalacturonans appear later during maturation [8]. On the contrary, in *fra2* cytokinetic cells, cell plates were enriched with demethylesterified homogalacturonans even at early cytokinesis, which was further intensified in post-cytokinetic cells. In parallel, although callose is universally present as a “solidifier” in expanding cell plates [8], its presence in *fra2* cytokinetic cells was significantly decreased. Taken together, the above findings indicate that, due to phragmoplast defects, the formation, consolidation, and expansion of the cell plate cannot keep pace with its maturation in dividing *fra2* root cells. Therefore, as a consequence of delayed cytokinesis [21], the ingredients of the cell plate are modified earlier than expected, i.e., during cell plate expansion than after its fusion with the parent walls.

The above claim is further supported by the distribution of KNOLLE syntaxin. In general, KNOLLE is present during the whole process of cytokinesis, also remaining temporarily at early interphase in the nascent cross wall [6,7]. Accordingly, its presence in newly formed cell walls of interphase cells, as observed in wild-type roots, is well-expected (Figure 8a). On the contrary, in *fra2* roots, interphase cells with a KNOLLE signal were remarkably scarce, in comparison to the wild type. This difference may be attributed to the delayed cytokinesis of *fra2* dividing cells: as cytokinesis takes longer than normal to be completed, the entrance of post-cytokinetic cells to interphase is also delayed. As a consequence, KNOLLE does not catch up with the cytokinesis/interphase transition and is depleted from new cell walls prematurely.

The main structural defects observed in *fra2* cell plates and cross walls were the increased and uneven thickness, presence of multilamellar bodies and large gaps through the whole cell plate surface. One reason for building an unevenly thick cell plate may be the increased length of phragmoplast microtubules. De Keijer et al. [9] have shown in *Physcomitrella patens* that when phragmoplast microtubules were excessively overlapping and their (+) ends penetrated through the expected mid-zone, a thick and irregularly shaped cell plate was built. In cytokinetic cells of *fra2* root, microtubules were also observed to penetrate through the whole cell plate plane (Figure A1 and Figure 6e), which may result in some of the malformations observed. In addition, the increased stability and persistence of microtubules in the so-called lagging phragmoplast zone [4] may result in excess vesicle transport and fusion at already consolidated cell plate areas, probably responsible for the formation of multilamellar bodies (Figure 5c). Moreover, a mechanical dislocation of cell plate parts, due to “pushing” by elongated phragmoplast microtubules, may account for the failure of those parts to make a proper junction (Figure 5a). An explanation why cell plate gaps persist and are sometimes inherited by nascent cell walls (Figure 6a–c), may be the early cell plate maturation, in comparison with its expansion, also combined with the lack of the stabilizing effect of callose. Gaps are created, because of phragmoplast defects, then fail to be healed, as cell plate matures and attains the properties of a cross wall (presence of demethylesterified homogalacturonans, low callose content) even before its eventual fusion with the parent walls. In support of such a view, perforated cell plates were prominent in callose-defective *massue* mutants of *A. thaliana* [22].

Moreover, the influence of defective preprophase microtubule band organization in p60-katanin mutants should not be overlooked. Preprophase bands in the above mutants are notoriously malformed, asymmetrically organized or even incomplete [19,20,21]. Consequently, division site demarcation and properties are expected to be compromised in *fra2* dividing cells. The pivotal role of the division site, pre-established by preprophase band organization, in proper cell plate maturation and flattening have been already reported [23]. It could be thus assumed that due to faulty preprophase band organization, the division site cannot exert its effect on cell plate “finishing” to a new cross wall.

In conclusion, phragmoplast defects due to p60-katanin malfunction result in faulty cell plate and cross wall formation during *fra2* cytokinesis. In particular, delayed phragmoplast expansion and increased microtubule length lead to a loss of synchronization between the growth and chemical maturation of the cell plate. Consequently, cell plates exhibit structural defects, such as uneven thickness and gaps, which are then bequeathed to the nascent cross walls. A challenge for further research is to investigate the effect of such cytokinetic abnormalities on specialized cell types, especially those occurring by asymmetric divisions, such as meristemoids and trichome initials.

## 4. Materials and Methods

Plant materials, namely seeds of wild type (Col-0) and the *fra2* p60-katanin *A. thaliana* mutant, were purchased from the Nottingham Arabidopsis Stock Center (NASC). All the chemicals and reagents used in this study were supplied by Sigma (Taufkirchen, Germany), Merck (Darmstadt, Germany), and Applichem (Darmstadt, Germany), and all the following steps were carried out at room temperature, unless stated otherwise.

Seeds of the wild-type and *fra2* were bleach-surface-sterilized and grown on solid ½ MS agar medium as previously described [24]. Roots of 5-day-old wild-type and *fra2* seedlings were prepared for transmission electron microscopy (TEM) by the protocol in [25]. In brief, root segments from at least 15 roots, comprising the meristematic root zone, were fixed for 4 h in 3% (*v/v*) glutaraldehyde in 50 mM sodium cacodylate, pH 7, post-fixed in 1% (*w/v*) osmium tetroxide in the same buffer for 3 h, dehydrated in an acetone series and embedded in Spurr’s resin. Ultrathin sections (70–90 nm), double stained with uranyl acetate and lead citrate, were observed with a JEOL JEM 1011 TEM at 80 kV and micrographs were acquired with a Gatan ES500 W camera. Root segments of both wild-type and *fra2* seedlings were also prepared for immunodetection of demethylesterified homogalacturonans (DeSPHGs) with the JIM5 rat antibody (Plant Probes, University of Leeds), while anti-*β*-1,3-glucan was conducted according to [26]. In short, root tips were fixed in 4% (*w/v*) paraformaldehyde in PEM buffer (50 mM PIPES, 5 mM EGTA, 5 mM MgSO_4_, pH 6.8) for 1 h. Fixed roots were rinsed twice in the same buffer for 10 min. Cell walls were digested for 1 h in 2% (*w/v*) Cellulase Onozuka R-10 (Duchefa, Haarlem, Netherlands) in PEM. Then, root tips were extracted with 5% (*v/v*) DMSO + 1% (*v/v*) Triton X-100 in PBS for 1 h. Incubations with JIM5, anti-β-1,3-glucan and FITC-anti-rat (Invitrogen, Carlsbad, CA), all diluted 1:40 in PBS, were carried out sequentially overnight in the dark with a washing intermediate step (3 × 10 min). Finally, after DNA counterstaining with DAPI and washing in PBS, the specimens were mounted in a PBS-glycerol mixture (1:2 *v/v*), supplemented with 0.5% (*w/v*) p-phenylenediamine as anti-fade agent, while in some cases the roots were gently squashed between the microscope slide and coverslip, to release some meristematic root cells from the surrounding tissues. *α*-tubulin and KNOLLE immunolabeling (anti-KNOLLE diluted 1:2000) was conducted as stated in [19]. The specimens were examined with a Zeiss LSM780 confocal laser scanning microscope (CLSM) as previously described [25] or with a Zeiss Axioplan microscope (Zeiss, Berlin, Germany), equipped with a Zeiss Axiocam MRc5 digital camera, using the ZEN 2.0 software, according to the manufacturer‘s instructions. Confocal, epi-fluorescence, and TEM images were processed with Adobe Photoshop with only linear settings.

The corrected total cell fluorescence (CTCF) intensity measurements were performed using the Image J (http://rsbweb.nih.gov/ij/) software according to [27]. For CTCF measurements for either callose or anti-KNOLLE immunostaining images, seven roots of wild type and seven of *fra2* were accessed and results were obtained from a total number of 1000 cells in each case. Cell counts exhibiting anti-KNOLLE signal in wild-type and *fra2* were expressed as percentage. Statistical analyses (ANOVA with Dunnett’s multiple comparison test) were performed using GraphPad software (San Diego, CA, USA), with significance at *p* < 0.05.

## Data Availability

Not applicable.

## Abbreviations

CLSM	Confocal laser scanning microscope
DeSHGs	Demethylesterified homogalacturonans
TEM	Transmission electron microscope
MAPs	Microtubule associated proteins
